# St Mark’s protocol for standardised examination under anaesthesia for rectovaginal fistulae

**DOI:** 10.1007/s10151-025-03257-6

**Published:** 2026-03-06

**Authors:** M. Okocha, A. Rowe, K. Elgendy, G. Thomas, P. Tozer, C. Vaizey

**Affiliations:** 1 Sir Alan Parks Physiology and Pelvic Floor Unit, St Mark’s the National Bowel Hospital, Central Middlesex Hospital, Acton Lane, London, NW10 7NS UK; 2 Institute of Continuing Education, University of Cambridge, Cambridge, UK; 3 Nottingham University Hospitals NHS Foundation Trust, Nottingham, UK; 4 Imperial College London, London, UK

**Keywords:** Rectovaginal fistula, obstretric anal sphincter injuries, endoanal ultrasound, proctology

## Abstract

Rectovaginal fistulae are uncommon but highly morbid, with presentations ranging from vaginal defaecation to subtle symptoms such as flatus, discharge, or recurrent infection. Imaging and contrast studies may be inconclusive for short, low-lying tracts, and ultrasound techniques can be limited by false-negative results. A structured examination under anaesthesia therefore remains central to definitive diagnosis and operative planning. The St Mark’s protocol standardises this assessment using a reproducible five-stage sequence: (1) direct proctovaginal inspection with evaluation of the rectovaginal septum, perineal body, and anterior sphincter complex; (2) intraoperative endoanal ultrasonography where expertise permits; (3) probing to confirm patency of small or occult openings; (4) insufflation with a vaginal bubble test to demonstrate communication, particularly for higher fistulae; and (5) rectal methylene blue dye testing with isolated vaginal swabbing to confirm persistence. This paper describes the protocol and its role in consistent documentation, anatomical characterisation, and informed intraoperative decision-making.

## Introduction

Rectovaginal fistulae (RVFs) represent one of the most distressing conditions in colorectal practice. Despite their low incidence, they impose a disproportionate psychological and physical burden on patients, impairing continence, intimacy, and quality of life [1, 2]. RVFs may arise from obstetric trauma, Crohn’s disease, pelvic surgery, radiation, or malignancy [2–4], and treatment can be complex [5, 6]. The symptomatology varies, with vaginal defaecation being the clearest symptom. More subtle presentations may include vaginal flatus, discharge, or thrush [6].

In the more subtle cases, diagnosis can be challenging. Cross-sectional imaging, while useful in assessing the extent and surrounding pathology, often fails to confirm short, low-lying fistulae. MRI remains the standard in Crohn’s disease and complex, e.g. post-radiation, fistulae, combined with an examination under anaesthesia [7, 8]. Similarly, contrast studies per rectum and vagina may be diagnostic but they do not exclude a fistula [9]. Enhanced-definition endoanal and transperineal ultrasound have gained traction for characterising sphincteric and perineal involvement, although availability and expertise vary widely. These modalities cannot be used to fully exclude a fistula, as both have a recognised false-negative rate [10].

Ultimately, definitive diagnosis and planning depend on clinical evaluation, often under anaesthesia. Examination under anaesthesia (EUA) enables systematic proctovaginal inspection, testing for tract patency, biopsy of suspicious areas, assessment of the integrity of the sphincters, and documentation of healing after conservative or surgical management. In equivocal cases, EUA serves not only to establish the presence of a fistula but also to reassure the patient when healing is confirmed. This is particularly important in cases where persistent symptoms do not reflect persistent pathology.

This paper documents the St Mark’s protocol for examination under anaesthesia (EUA) in suspected rectovaginal fistulae. The five-stage approach—inspection, intraoperative endoanal ultrasonography (where local expertise permits), probing, insufflation, and dye testing—provides a reproducible framework for diagnosis and operative decision making, whether performed before repair or to assess healing.

## Technique

The patient is admitted as a day case. Under general anaesthesia, they are positioned in lithotomy. The protocol consists of five key stages:

### Stage 1: Direct visual inspection

**Fig. 1:**
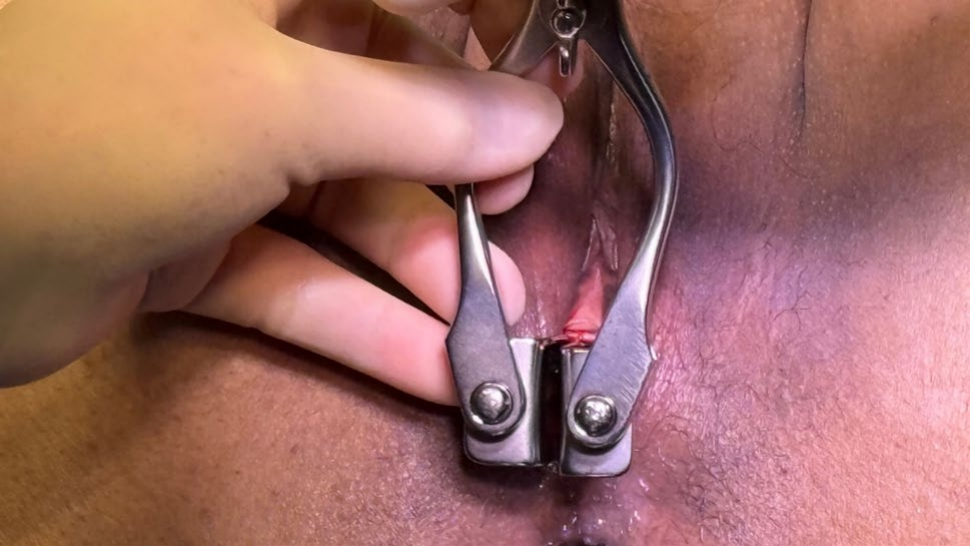
Parks retractor and inspection of rectovaginal septum

Using a Parks retractor vaginally and an Eisenhammer retractor rectally, the rectovaginal septum and perineum are thoroughly examined (as in Fig. 1). The operator inspects for fistulous openings, granulation tissue, mucosal ulceration, Crohn’s-related changes, or obstetric scarring. In addition, the perineal body and labia are assessed for volume and allowance of a potential Martius flap. The integrity and symmetry of the anterior sphincter complex are evaluated.

### Stage 2: Intraoperative endoanal ultrasonography (where local expertise permits)

**Fig. 2:**
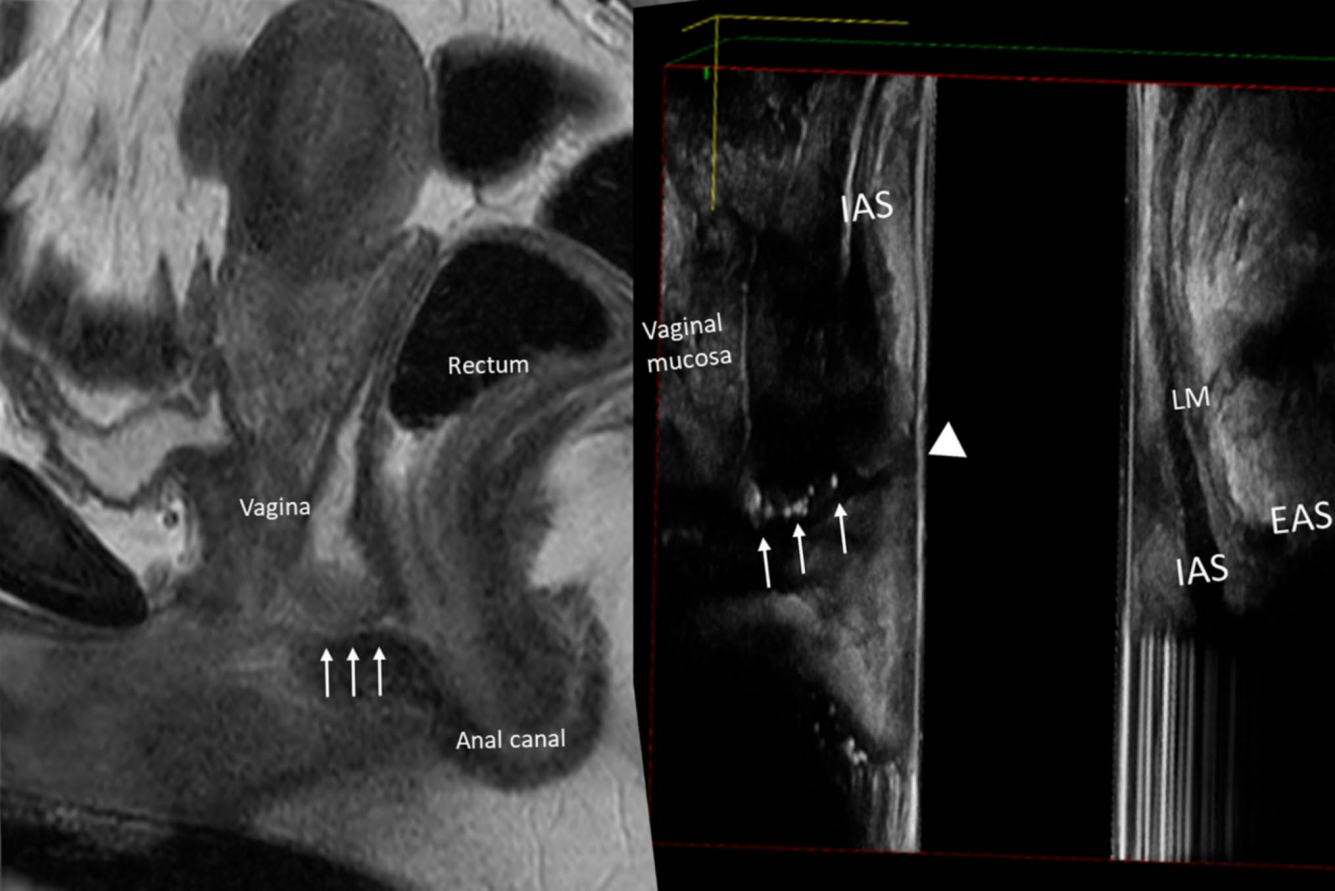
Comparison of preoperative MRI (left) and intraoperative endoanal ultrasound (right) demonstrating concordant fistula tract (arrows) between the anal canal and posterior vaginal wall. *IAS* internal anal sphincter, *EAS* external anal sphincter, *LM* longitudinal muscle

Immediately following inspection, intraoperative endoanal ultrasonography may be performed to delineate sphincteric integrity, define the fistula tract, and correlate findings with preoperative MRI. A 3D endoanal probe enables identification of occult or secondary extensions and assessment of the degree of fibrosis or sphincteric disruption, particularly in recurrent, high, or anterior fistulae. Figure 2 shows intraoperative EAUS findings compared with preoperative MRI.

### Stage 3: Use of a fistula probe to define smaller openings

Lockhart-Mummery or lacrimal probes are used to demonstrate patency of fistulae not apparent on inspection.

### Stage 4: Insufflation and bubble test

**Fig. 3:**
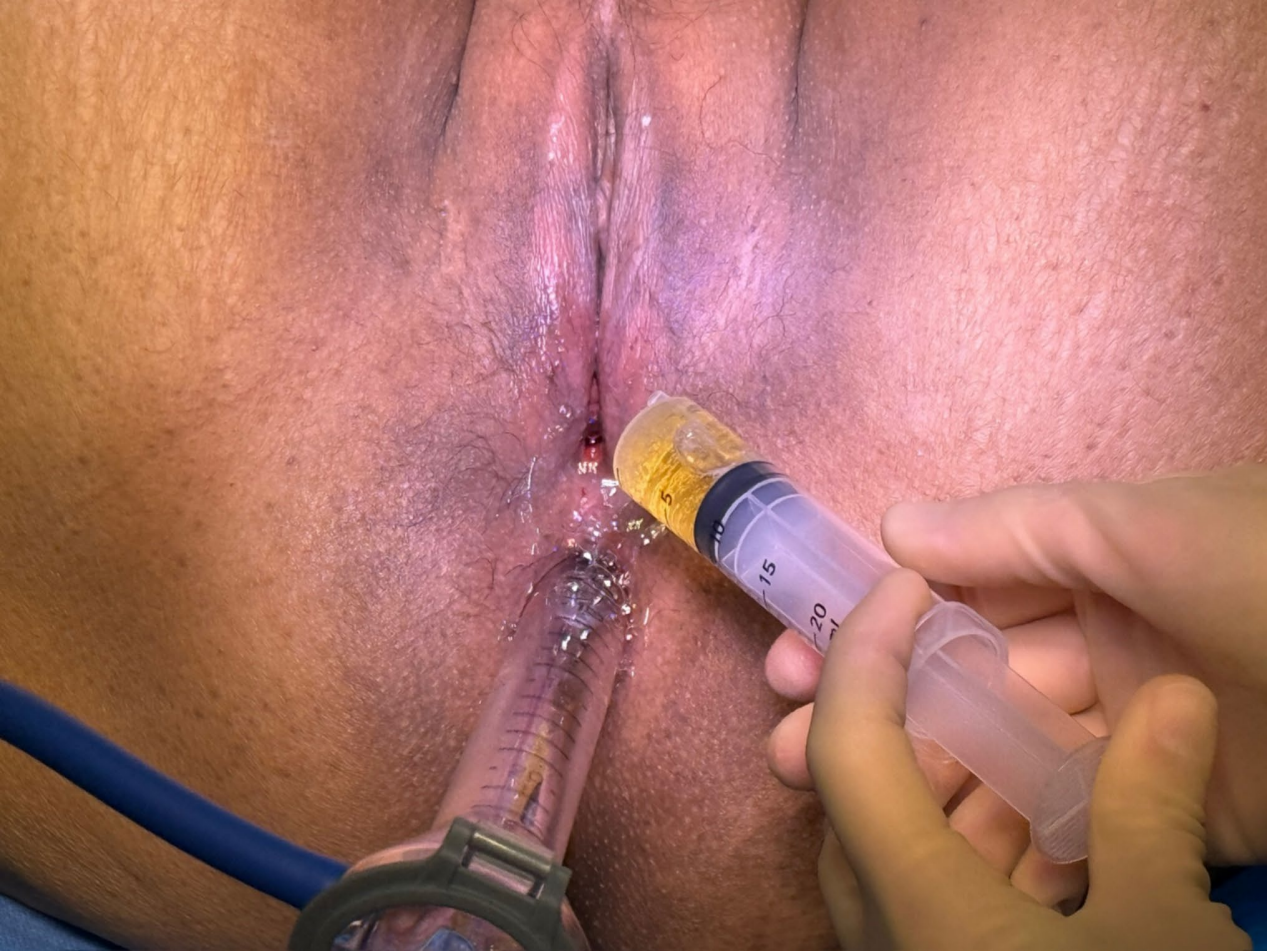
Positive bubble test

The patient is temporarily put in the Trendelenburg position. Chlorhexidine acetate with cetrimide irrigation solution is introduced into the vaginal canal. A rigid sigmoidoscope is inserted into the rectum, and air insufflation is performed. Observation of bubbles in the vaginal fluid confirms fistulous communication (Fig. 3). This test can be particularly useful for high fistulae up in the fornices.

### Stage 5: Methylene blue dye test

**Fig. 4:**
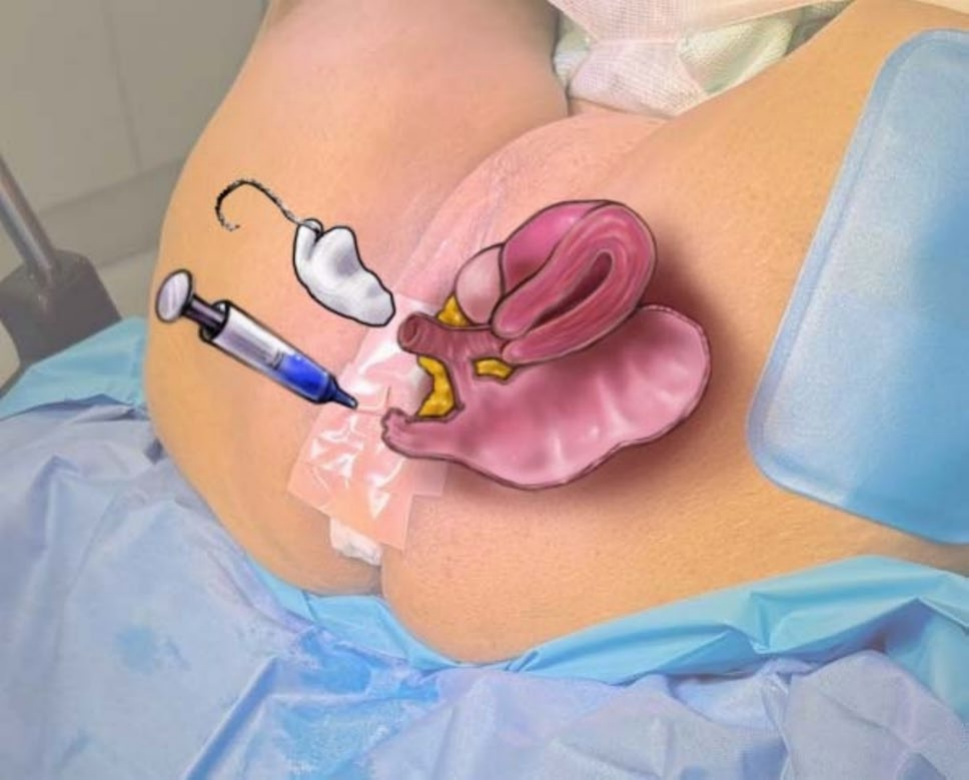
Medical illustration of relevant anatomy and patient position

**Fig. 5:**
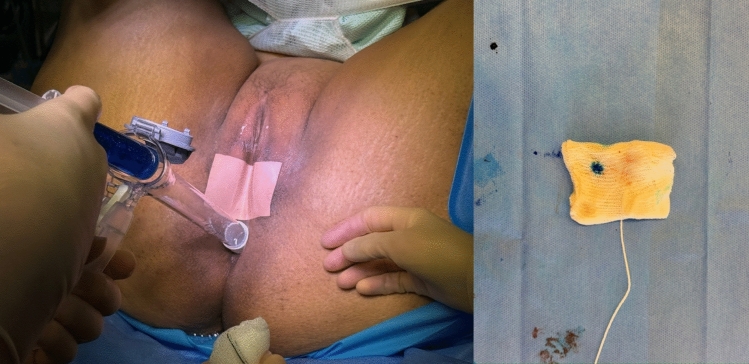
Methylene blue dye test and swab after positive result

A sterile throat pack with a string tail is inserted into the vaginal canal, and the introitus is sealed with waterproof Hy-Tape™. A dilute solution of methylene blue (5 ml 1% in 200 ml saline) is introduced rectally, as demonstrated in Fig. 4. The swab is removed after 30 min, with care not to allow cross-contamination. Blue staining indicates passage of dye and confirms a persistent fistula, as in Fig. 5.

## Conclusion

This five-stage protocol provides a structured and reproducible approach to the assessment of rectovaginal fistulae under anaesthesia. It is particularly valuable in patients with ambiguous imaging, persistent non-specific symptoms, or suspected healing after repair. Incorporation of intraoperative endoanal ultrasonography, where expertise allows, enhances anatomical definition and correlation with preoperative imaging. The protocol enables comprehensive documentation, with photographic or video capture where appropriate, and facilitates informed intraoperative decision making, including progression to definitive repair when indicated.

## Data Availability

No datasets were generated or analysed during the current study.
